# Rapid, efficient auxin-inducible protein degradation in *Candida* pathogens

**DOI:** 10.1128/msphere.00283-23

**Published:** 2023-08-18

**Authors:** Kedric L. Milholland, Justin B. Gregor, Smriti Hoda, Soledad Píriz-Antúnez, Encarnación Dueñas-Santero, Bao Gia Vu, Krishna P. Patel, W. Scott Moye-Rowley, Carlos R. Vázquez de Aldana, Jaime Correa-Bordes, Scott D. Briggs, Mark C. Hall

**Affiliations:** 1 Department of Biochemistry, Purdue University, West Lafayette, Indiana, USA; 2 Department of Biomedical Sciences, Universidad de Extremadura, Badajoz, Spain; 3 Institute of Functional Biology and Genomics, Consejo Superior de Investigaciones Científicas (CSIC), Universidad de Salamanca (USAL), Salamanca, Spain; 4 Department of Molecular Physiology and Biophysics, Carver College of Medicine, University of Iowa, Iowa City, Iowa, USA; 5 Institute for Cancer Research, Purdue University, West Lafayette, Indiana, USA; University of Georgia, Athens, Georgia, USA

**Keywords:** auxin-inducible degradation, *Candida albicans*, *Candida glabrata*, Cdc14, Gcn5, Mob2, targeted protein degradation

## Abstract

A variety of inducible protein degradation (IPD) systems have been developed as powerful tools for protein functional characterization. IPD systems provide a convenient mechanism for rapid inactivation of almost any target protein of interest. Auxin-inducible degradation (AID) is one of the most common IPD systems and has been established in diverse eukaryotic research model organisms. Thus far, IPD tools have not been developed for use in pathogenic fungal species. Here, we demonstrate that the original AID and the second generation, AID2, systems work efficiently and rapidly in the human pathogenic yeasts, *Candida albicans* and *Candida glabrata*. We developed a collection of plasmids that support AID system use in laboratory strains of these pathogens. These systems can induce >95% degradation of target proteins within minutes. In the case of AID2, maximal degradation was achieved at low nanomolar concentrations of the synthetic auxin analog 5-adamantyl-indole-3-acetic acid. Auxin-induced target degradation successfully phenocopied gene deletions in both species. The system should be readily adaptable to other fungal species and to clinical pathogen strains. Our results define the AID system as a powerful and convenient functional genomics tool for protein characterization in fungal pathogens.

**IMPORTANCE:**

Life-threatening fungal infections are an escalating human health problem, complicated by limited treatment options and the evolution of drug resistant pathogen strains. Identification of new targets for therapeutics to combat invasive fungal infections, including those caused by *Candida* species, is an urgent need. In this report, we establish and validate an inducible protein degradation methodology in *Candida albicans* and *Candida glabrata* that provides a new tool for protein functional characterization in these, and likely other, fungal pathogen species. We expect this tool will ultimately be useful for the identification and characterization of promising drug targets and factors involved in virulence and drug resistance.

## INTRODUCTION

Historically, functional characterization of proteins has largely depended either on their biochemical isolation and analysis, or on gene inactivation through random mutation or targeted chromosome editing followed by phenotypic observation. However, permanent gene inactivation can significantly alter cellular physiology, including activating adaptation mechanisms or selecting for compensatory mutations. More recently, transcriptional repression and RNA interference technologies have provided useful alternatives, for example, in studying essential gene products (1 – 5). Transcriptional repression and RNA silencing methods are often slow-acting, particularly for stable proteins that must be fully degraded before phenotypes appear. In these cases, the observed phenotypes may not be directly linked to functions of the protein of interest but rather an indirect consequence of its persistent loss of function. Moreover, the permanent or slow-acting nature of these methods makes them less applicable for mechanistic studies of dynamic cellular processes. The use of specific chemical inhibitors is an ideal way to study protein function that circumvents many of the problems associated with molecular genetic methods for reducing protein function. Inhibitors can be fast-acting, reversible, and highly specific, often achieving near-complete loss of function. However, not all proteins have functions like enzymatic activity that can be readily inhibited by small molecules, and effective, highly specific inhibitors are not available for most proteins.

**Fig 1:**
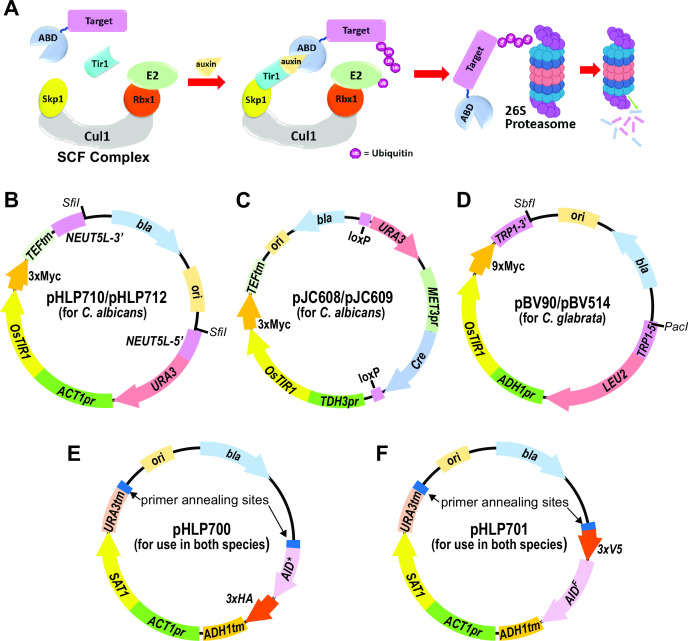
Overview of AID system and constructs for *Candida* strain engineering. (A) AID system function requires the following: (i) fusion of an ABD, e.g., from *Arabidopsis thaliana* IAA17, to a target protein and (ii) ectopic expression of a plant *TIR1* gene, e.g., from *Oryza sativa*, that can interact with the host organism’s endogenous SCF E3 ubiquitin ligase complex. Addition of auxin to cells induces Tir1-ABD interaction and recruitment of the target protein to the SCF-E2 complex for polyubiquitination and subsequent degradation via the 26S proteasome. (B) Plasmid containing *OsTIR1* integration cassette for *Candida albicans*. Cassette bounded by *NEUT5L* homology regions can be excised by *SfiI* digest or amplified by PCR for transformation and integration at *NEUT5L* locus. Alternatively, PCR amplification of the cassette can be designed for integration at other loci with the appropriate homology regions. (C) Plasmid containing *OsTIR1* integration cassette with recyclable *URA3* marker. After integration of PCR-amplified cassette at any desired locus, removal of methionine leads to Cre expression and recombination between loxP sites, excising *URA3* and *Cre* genes. (D) Plasmid containing *OsTIR1* integration cassette for *Candida glabrata*. Cassette bounded by *TRP1* homology regions can be excised by *SbfI/PacI* restriction digest or amplified by PCR for transformation and integration at *TRP1*. (E and F) Plasmid templates for C-terminal target tagging with either AID*/3xHA (E) or 3xV5/AID^F^ (F) degrons. Integration cassettes are amplified by PCR using the designated primer annealing sites and primers containing homology regions for the 3ʹ end of the target gene. pr: promoter; tm: terminator; *bla:* beta lactamase gene encoding ampicillin resistance; ori: *Escherichia coli* origin of replication.

The advent of inducible protein degradation (IPD) technologies has provided a “best of both worlds” option for protein functional characterization. In IPD systems, a target gene is engineered using molecular genetic tools so that the encoded protein is functional and expressed at a natural level but its proteolytic degradation can be rapidly triggered by an external stimulus. These systems can be applied, in principle, to any target protein yet have the speed, specificity, and efficacy of chemical inhibitors. Several inducible degradation systems have been developed for use in a variety of model organisms (6, 7). One of the most popular is the auxin-inducible degradation (AID) system (Fig. 1A), based on the natural mode of action of the plant hormone, auxin. Auxins act as molecular glues that promote the physical association of auxin-binding domain (ABD) proteins with the Skp1-Cul1-F-box protein (SCF)^Tir1^ ubiquitin ligase (8 – 10). This results in polyubiquitination of the ABD protein and its subsequent recognition and proteolysis via the 26S proteasome. In 2009, Kanemaki and colleagues demonstrated that fusion of a target gene to the coding sequence of an ABD and expression of a plant Tir1 F-box protein in *Saccharomyces cerevisiae* or cultured human cells allowed rapid degradation of the fusion protein simply by addition of the natural auxin, 3-indoleacetic acid (IAA) to the culture medium (11).

The AID system depends on the ability of plant Tir1 to interact with the endogenous core SCF ubiquitin ligase of the target organism, and on the availability of tools to (i) genomically tag the gene of interest with an ABD sequence and (ii) stably express the plant *TIR1* gene. To date, inducible target protein degradation using the AID system has been validated in several fungal, protozoan, and metazoan species, including *S. cerevisiae* (11)*, Schizosaccharomyces pombe* (12)*, Caenorhabditis elegans* (13), *Drosophila melanogaster* (14)*,* mammalian oocytes (15), human cell culture (16), mice (17), the protozoan pathogens *Toxoplasma gondii* (18) and *Plasmodium falciparum* (19), and the industrial yeast *Yarrowia lipolytica* (20). A limitation of the original AID system is the relatively high concentration of auxin required to induce degradation (typically high micromolar to low millimolar), which was shown to have physiological effects (21, 22) or toxicity (17) in some systems. A recently developed second generation AID system, named AID2, greatly reduces the potential for toxicity or non-specific physiological consequences. AID2 was inspired by the structure-guided engineering of Tir1 to bind the larger, synthetic auxin analogs 5-phenyl-IAA and 5-adamantyl-IAA in plants (23). Replacing wild-type Tir1 with the engineered mutant in AID systems provided efficient target degradation at synthetic auxin concentrations orders of magnitude lower than natural IAA (17, 24).

IPD systems, including AID, have not been established in human fungal pathogens, where they would be valuable for functional characterization of proteins to better understand pathogen biology, virulence mechanisms, drug resistance, and other clinically relevant processes. Moreover, they could be useful for antifungal drug development. Invasive fungal infections kill an estimated 1.7 million people each year (25) and fungal pathogens are developing resistance to the few available antifungal drug classes (26). As a result, there is significant interest in understanding pathogen infection and drug resistance mechanisms. Here, we demonstrate that the AID system works efficiently and rapidly in the human pathogenic yeasts *Candida albicans* and *Candida glabrata. Candida* species are major nosocomial pathogens of immune-compromised individuals, accounting for nearly 20% of hospital-acquired bloodstream infections worldwide, with high mortality rates (27, 28). The opportunistic commensal organisms, *C. albicans* and *C. glabrata,* account for the most and second-most, respectively, invasive *Candida* infections. The World Health Organization designated *C. albicans* as one of four critical priority group species in its 2022 report on priority fungal pathogens, while *C. glabrata* was designated as one of seven high-priority group species (29). We, therefore, developed reagents for using the original AID and the second generation AID2 systems in lab strains of *C. albicans* and *C. glabrata* as a new tool for characterizing protein function in these problematic pathogens. Our work provides a blueprint for expanding this system to clinical isolates and other pathogen species in the future.

## RESULTS and DISCUSSION

### Engineering AID and AID2 systems for use in *C. albicans* and *C. glabrata*

We designed integration vectors for expression of *Oryza sativa TIR1* (*OsTIR1*) to be used in common lab strains of *C. albicans* and *C. glabrata* to assess performance of the AID system in these pathogens. For *C. albicans*, we synthesized a codon-optimized *OsTIR1* gene with 3xMyc epitope tag and constructed a plasmid with a restriction enzyme-excisable cassette for integration at the *NEUT5L* locus (30) with *URA3* selectable marker and *OsTIR1* expression driven by the *ACT1* promoter (Fig. 1B). We also designed a recyclable, PCR-amplifiable *OsTIR1* integration cassette with *URA3* marker using a previously developed Cre-lox-based *C. albicans* vector system (31), in which *OsTIR1* is expressed from the *TDH3* promoter (Fig. 1C). For *C. glabrata,* we constructed a plasmid with excisable cassette for integration at the *TRP1* locus with *LEU2* selectable marker and *OsTIR1-9xMyc* expressed from the *ADH1* promoter (Fig. 1D). We then introduced the F74A codon change in the *OsTIR1* coding sequence of all plasmids to allow the use of the synthetic auxin analog 5-adamantyl-indole-3-acetic acid (5-Ad-IAA) and the AID2 system (17, 24).

We designed integration cassettes for C-terminal degron tagging of target proteins for use in both *C. albicans* and *C. glabrata*, and inserted them into plasmid backbones to serve as templates for PCR amplification. One cassette contains the coding sequence for the full 229 amino acid degron domain of the *A. thaliana* IAA17 auxin-binding protein (11), hereby designated AID^F^, fused to a 3xV5 epitope. The second contains a smaller truncation of the IAA17 degron region (aa 71-114), previously named AID* (32) fused to a 3xHA epitope. In each case, the degron is followed by a stop codon, transcriptional terminator, and CTG clade codon-optimized *SAT1* marker gene (33) providing nourseothricin resistance for positive selection (Fig. 1E and F). AID strain construction with these vectors requires two genome integration steps: (i) integration of the *OsTIR1* expression cassette generated by restriction digest or PCR amplification, and (ii) integration of a PCR-amplified degron tag at the 3ʹ end of the desired target gene. For diploid species like *C. albicans* both alleles can be tagged, or one can be deleted.

### AID and AID2 systems provide robust, rapid target degradation in *C. albicans* and *C. glabrata*

**Fig 2:**
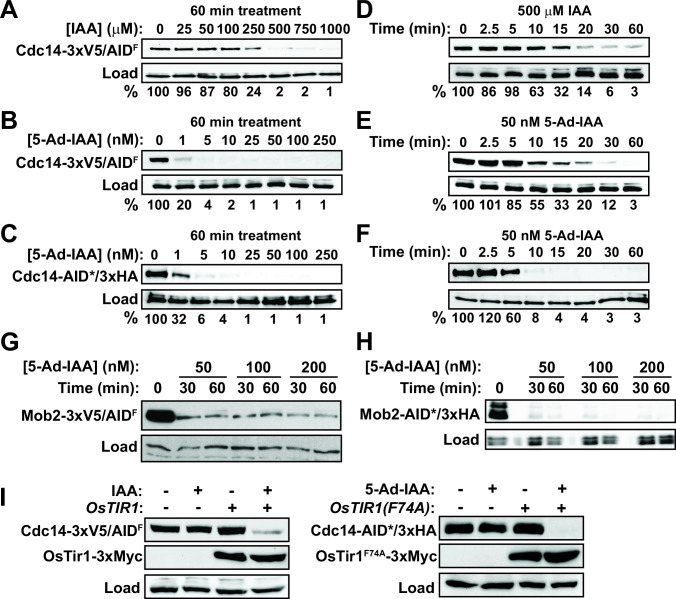
AID technology provides rapid, efficient target degradation in *C. albicans*. (A) Cdc14-3xV5/AID^F^ degradation in cells expressing wild-type OsTir1 (HCAL125) was measured by anti-V5 immunoblotting after treatment of log-phase liquid yeast extract, peptone, dextrose (YPD) cultures with the indicated IAA concentrations for 60 min. Percent protein remaining relative to the untreated culture was quantified by digital imaging. (B) Same as panel (A) using cells expressing OsTir1^F74A^ (HCAL126) and treated with varying 5-Ad-IAA concentrations. (C) Same as panel (B) measuring degradation of Cdc14-AID*/3xHA (HCAL128) with anti-HA antibody. (D) Time dependence of Cdc14-3xV5/AID^F^ degradation in HCAL125 cells from panel (A) treated with 500 µM IAA measured by anti-V5 immunoblotting. Percent protein remaining relative to time = 0 was quantified by digital imaging. (E) Time dependence of Cdc14-3xV5/AID^F^ degradation in HCAL126 cells from panel (B) treated with 50 nM 5-Ad-IAA. (F) Time dependence of Cdc14-AID*/3xHA degradation in HCAL128 cells from panel (C) treated with 50 nM 5-Ad-IAA measured by anti-HA immunoblotting. (G) Degradation of Mob2-3xV5/AID^F^ at the indicated times after 5-Ad-IAA treatment in a strain expressing *OsTIR1^F74A^* from the *TDH3* promoter (OL3372) was monitored by anti-V5 immunoblotting. Anti-Cdc11 was used as a loading control. (H) Same as (A), monitoring degradation of Mob2-AID*/3xHA in a strain expressing *OsTIR1^F74A^* from the *ACT1* promoter (OL3309). Anti-PSTAIR was used as a loading control. (I) The dependence of Cdc14-3xV5/AID^F^ degradation on OsTir1 and IAA (left, strains HCAL112 and HCAL125) and the dependence of Cdc14-AID*/3xHA degradation on OsTir1^F74A^ and 5-Ad-IAA (right, strains HCAL113 and HCAL128) were determined by immunoblotting with anti-V5 and anti-HA immunoblotting, respectively. Log-phase cultures were treated with 500 µM IAA or 50 nM 5-Ad-IAA or mock treated with an equal volume of dimethyl sulfoxide (DMSO) for 60 min prior to harvesting. In all panels except (G), PSTAIR (Cdc28) was used as a load control. In panel (G), Cdc11 was used as a load control.

For initial testing and comparison of the AID and AID2 systems in *C. albicans*, we tagged the Cdc14 phosphatase at its C-terminus in heterozygous *CDC14/cdc14Δ* strains expressing either wild-type OsTir1 or OsTir1^F74A^. Reductions in Cdc14 activity render *C. albicans* hypersensitive to cell wall stress and impair hyphal development, allowing facile phenotypic monitoring of AID system performance (34, 35). Moreover, Cdc14 has been successfully targeted using AID in *S. cerevisiae* (36, 37). We first measured Cdc14-3xV5/AID^F^ degradation by immunoblotting after 60-min treatment with varying concentrations of IAA in strains expressing wild-type OsTir1, or the synthetic auxin 5-Ad-IAA in strains expressing OsTir1^F74A^. In both the cases, Cdc14 level was consistently reduced to ~1% of its untreated steady-state level (Fig. 2A and B; Fig. S1A and B). With the original AID system, achieving >95% reduction in Cdc14 level required 500 µM IAA, whereas with the AID2 system >95% reduction was achieved at 100,000-fold lower auxin concentration (5 nM 5-Ad-IAA). Using the AID2 system, we then compared the effectiveness of the larger 3xV5/AID^F^ degron and the smaller AID*/3xHA degron. Maximal Cdc14 reduction and sensitivity to 5-Ad-IAA concentration were similar with the two degrons (Fig. 2B and C; Fig. S1B and C). Next, we measured the kinetics of Cdc14 degradation in *C. albicans* treated with auxin concentrations that reduced Cdc14 level >95%. The kinetics for the AID and AID2 systems and for the two different degron tags were similar with half-lives ranging from 5 to 15 min (Fig. 2D through F; Fig. S1D through F).

We also evaluated the *C. albicans* AID2 system on a different target, the Cbk1 kinase accessory protein, Mob2 (38, 39). Mob2-3xV5/AID^F^ was robustly degraded within 30 min of 50 nM 5-Ad-IAA addition in a strain expressing *OsTIR1^F74A^* from the *TDH3* promoter, generated with the recyclable *URA3* marker (Fig. 2G). Mob2-AID*/3xHA was degraded with equal success in a strain expressing *OsTIR1^F74A^* from the *ACT1* promoter (Fig. 2H). The extent of Mob2 degradation was comparable with the two *OsTIR1^F74A^* expression cassettes.

Finally, we measured the dependence of Cdc14-3xV5/AID^F^ stability on the presence of both OsTir1 and auxin, as auxin-independent target degradation has been reported in some systems (17, 32, 40). We did not observe auxin-independent target degradation in *C. albicans*. Detectable Cdc14 degradation required the presence of both OsTir1 and auxin in both the AID and AID2 systems (Fig. 2I).

**Fig 3:**
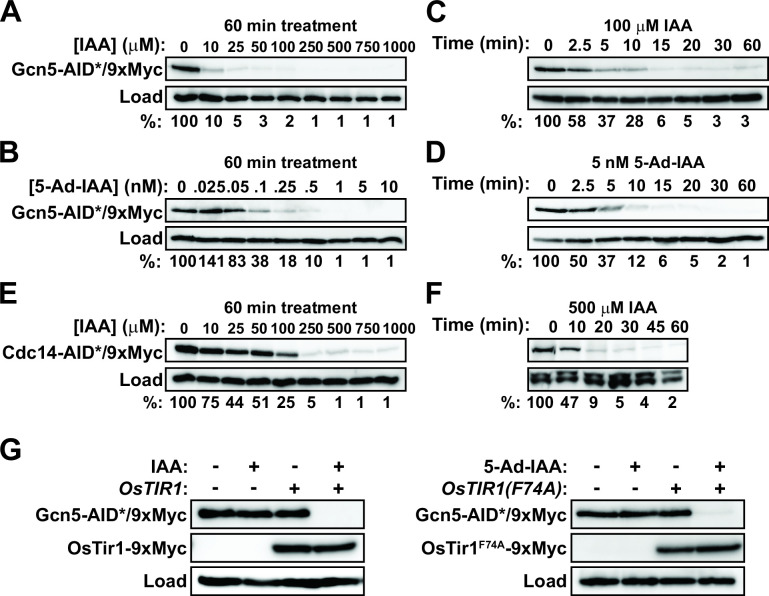
AID technology provides rapid, efficient target degradation in *C. glabrata*. (A) Gcn5-AID*/9xMyc degradation in cells expressing wild-type OsTir1 (SDBY1700) was measured by anti-Myc immunoblotting after treatment of log-phase liquid synthetic complete (SC) cultures with the indicated IAA concentrations for 60 min. Percent protein remaining relative to the untreated culture was quantified by digital imaging. (B) Same as panel (A), measuring Gcn5-AID*/9xMyc degradation in cells expressing OsTir1^F74A^ (SDBY1701) after treatment with the indicated concentrations of 5-Ad-IAA. (C) Time dependence of Gcn5-AID*/9xMyc degradation in SDBY1700 cells from panel (A) treated with 100 µM IAA was measured by anti-Myc immunoblotting. Percent protein remaining relative to time = 0 was quantified by digital imaging. (D) Time dependence of Gcn5-AID*/9xMyc degradation in SDBY1701 cells from panel (B) treated with 5 nM 5-Ad-IAA. (E) Same as panel (A) measuring Cdc14-AID*/9xMyc degradation in cells grown in YPD and expressing wild-type OsTir1 (SDBY1703). (F) Same as panel (C) measuring time dependence of Cdc14-AID*/9xMyc degradation in SDBY1703 cells grown in YPD. (G) The dependence of Gcn5-AID*/9xMyc degradation on OsTir1 and IAA (left, strains SDBY1700 and SDBY1702) and on OsTir1^F74A^ and 5-Ad-IAA (right, strains SDBY1701 and SDBY1702) were determined by anti-Myc immunoblotting. Log-phase cultures were treated with 100 µM IAA or 5 nM 5-Ad-IAA or mock treated with an equal volume of DMSO for 60 min prior to harvesting. In all panels except (F), histone H3 was used as a load control. In panel (F), PSTAIR (Cdc28) was used as a load control.

Similar results were observed when AID and AID2 systems were tested in *C. glabrata*. We tagged the histone acetyltransferase, Gcn5, and Cdc14 phosphatase with an AID*/9xMyc degron (32) in a strain expressing wild-type OsTir1. We also tagged Gcn5 in a strain expressing the AID2 variant OsTir1^F74A^. Gcn5 has also been successfully targeted using AID in *S. cerevisiae* (36). Steady-state Gcn5-AID*/9xMyc was reduced to ~1% of the initial levels in log-phase cultures, similar to Cdc14 in *C. albicans* (Fig. 3A and B; Fig. S2A and B). More than 95% reduction required 50 µM IAA in the presence of wild-type OsTir1 and 1 nM 5-Ad-IAA in the presence of OsTir1^F74A^. The kinetics of degradation after treatment with an auxin concentration sufficient for >95% degradation were similar in the AID and AID2 systems and consistent with Cdc14 in *C. albicans* (Fig. 3C and D; Fig. S2C and D). Cdc14-AID*/9xMyc degradation in *C. glabrata* was also rapid and efficient, requiring slightly lower IAA concentration than Cdc14-AID*/3xHA in *C. albicans* and exhibiting similar kinetics (Fig. 3E and F). Similar to *C. albicans*, detectable degradation of Gcn5-AID*/9xMyc in *C. glabrata* was dependent on the presence of both Tir1 and auxin (Fig. 3G).

We conclude that both the original AID system and the new AID2 system work effectively to achieve rapid and near-complete target protein loss in *C. albicans* and *C. glabrata* and are, therefore, likely to be useful tools for protein functional characterization in these species.

### IAA and 5-Ad-IAA have minimal impact on *C. albicans* and *C. glabrata* physiology

Natural auxin can impact cellular physiology in some systems, including *S. cerevisiae* (21, 22) and mice (17). We, therefore, assessed IAA and 5-Ad-IAA effects on *C. albicans* and *C. glabrata* growth rate and sensitivity to diverse stress conditions. For *C. albicans*’ liquid growth assays, we used 1 mM IAA and 1 µM 5-Ad-IAA concentrations, well above the concentrations needed for efficient target degradation. With or without integrated *OsTIR1*, growth was unaffected by the presence of IAA or 5-Ad-IAA (Fig. S3A). In serial dilution spotting assays on YPD agar plates supplemented with oxidative (H_2_O_2_), genotoxic [methyl methanesulfonate (MMS)], or osmotic (NaCl) stresses, and azole or echinocandin antifungal drugs, the presence of IAA and 5-Ad-IAA had no detectable effect on *C. albicans* and *C. glabrata* cell viability or growth rate (Fig. S3B and C). Furthermore, in *C. albicans*, 50 nM 5-Ad-IAA had no impact on *C. albicans* hyphal development induced by serum at 37°C in liquid cultures (Fig. S3D). Finally, 1 mM IAA and 1 µM 5-Ad-IAA had no impact on *C. albicans* and *C. glabrata* susceptibility to fluconazole and caspofungin in conventional liquid MIC (minimum inhibitory concentration) assays (Fig. S3E and data not shown). These results indicate that IAA and 5-Ad-IAA are mostly innocuous to *C. albicans* and *C. glabrata*. The very low concentration of 5-Ad-IAA required for target degradation relative to IAA makes it a particularly attractive option for minimizing non-specific physiological effects.

### AID2 phenocopies gene deletions in *C. albicans* and *C. glabrata*

**Fig 4:**
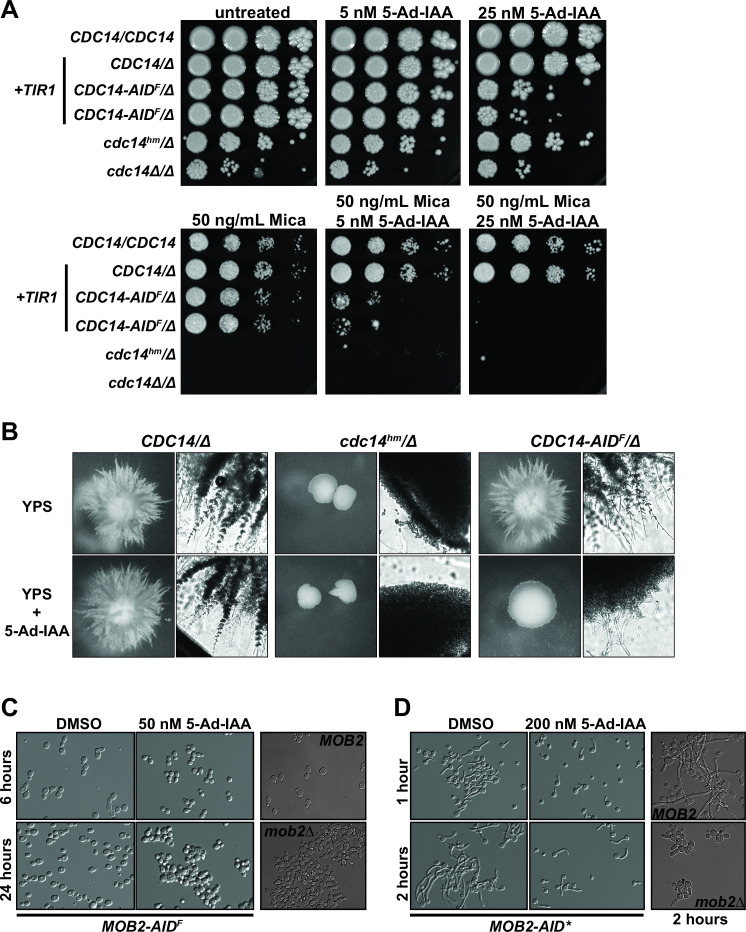
AID effectively phenocopies loss-of-function mutations in *C. albicans*. (A) Liquid cultures of *C. albicans* strains with the indicated *OsTIR1^(F74A)^* and *CDC14* genotypes (JC2712, HCAL111, HCAL126, HCAL102, and JC2711 from top to bottom) were serially diluted and spotted on YPD agar plates supplemented with micafungin and/or 5-Ad-IAA, as indicated. Plates were grown at 30°C for 3 days prior to imaging. The two *CDC14-AID^F^/Δ* samples (HCAL126) are independent transformants from the degron tag integration. The *cdc14^hm^* allele is our previously characterized hypomorphic mutant with reduced catalytic activity (35). (B) *C. albicans* strains with the indicated *CDC14* genotypes (JC2721, HCAL102, and HCAL126 from left to right) were grown embedded in yeast extract, peptone, sucrose (YPS) agar with or without 150 nM 5-Ad-IAA at 30°C for 4 days and colonies imaged with a dissecting scope (left) or Cytation 1 imaging plate reader with 4× brightfield objective (right). Fields of view for each imaging method are equivalent across all strains. (C) Differential interference contrast (DIC) images of exponentially growing *MOB2-3xV5/AID^F^* cells expressing *OsTIR1^F74A^* from the *TDH3* promoter (OL3372) incubated in YPD with or without 50 nM 5-Ad-IAA at 28°C for 6 h and 24 h. Images of exponentially growing BWP17 (wild-type *MOB2*) and *mob2Δ* derivative are shown for comparison. (D) DIC images of MOB2-AID*/3xHA cells expressing *OsTIR1^F74A^* from the *ACT1* promoter (OL3309) grown under hypha-inducing conditions (YPD + 10% serum at 37°C) in the presence or absence of 200 nM 5-Ad-IAA. Identically induced BWP17 (wild-type *MOB2*) and *mob2Δ* derivative at 2 h are shown for comparison.

For AID to be useful, target degradation should be extensive enough to induce loss-of-function phenotypes similar to gene deletions. Loss of Cdc14 function renders *C. albicans* hypersensitive to cell wall stresses, including echinocandin drugs, and also prevents hyphal development on agar plates (35). We used these phenotypes to compare the effects of AID2-mediated Cdc14 depletion to permanent loss-of-function mutations, including homozygous *CDC14* gene deletion and the catalytically impaired *cdc14^hm^* hypomorphic allele (35). Inclusion of 25 nM 5-Ad-IAA alone in YPD agar plates reduced the growth and viability of *C. albicans CDC14-3xV5/AID^F^* similar to *cdc14Δ/Δ*, demonstrating that AID also works efficiently in solid media (Fig. 4A). Supplementing YPD agar with 50 ng/mL micafungin alone had no impact on *CDC14-3xV5/AID^F^* cells compared to wild type, but was lethal to *cdc14^hm^/Δ* and *cdc14Δ/Δ*, demonstrating that the AID* degron tag does not significantly compromise Cdc14 function. Importantly, exposing *CDC14-3xV5/AID^F^* cells to just 5 nM 5-Ad-IAA in the presence of 50 ng/mL micafungin severely impaired growth, and 25 nM 5-Ad-IAA with micafungin eliminated growth completely.

Growth of *C. albicans* embedded within YPS agar medium, or on the surface of Spider agar medium, results in extensive hyphal development, leading to a filamentous colony morphology (Fig. 4B; Fig. S4). Reduced Cdc14 function completely prevents growth of radial hyphae both in embedded YPS agar and on Spider agar. *CDC14-3xV5/AID^F^* cells formed filamentous colonies like wild-type *CDC14* strains on both plate types in the absence of auxin. In contrast, supplementation of YPS or Spider plates with 5-Ad-IAA severely impaired hyphal filament development, similar to *cdc14^hm^/Δ* and *cdc14Δ/Δ*.

Deletion of *MOB2* results in a cell separation defect in *C. albicans* (38, 39). Including 5-Ad-IAA in liquid *MOB2-3xV5/AID^F^* cultures caused the same cell separation failure reported for *mob2Δ* cells (Fig. 4C). Cbk1-Mob2 activity is required for maintenance of hyphal growth (38, 39). Inclusion of 5-Ad-IAA in YPD-serum liquid medium at 37°C, conditions that strongly induce hyphae, compromised maintenance of hyphal growth in MOB2-AID*/3xHA cells, consistent with loss of Mob2 function (Fig. 4D).

**Fig 5:**
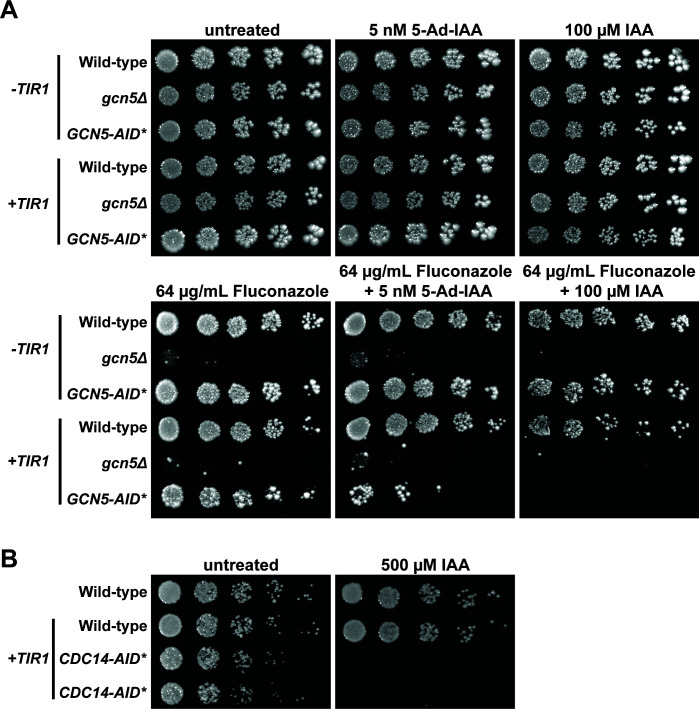
AID effectively phenocopies loss-of-function mutations in *C. glabrata*. (A) Liquid cultures of *C. glabrata* strains with the indicated *OsTIR1* and *GCN5* genotypes (row 1: KKY2001; row 2: SDBY1704; row 3: SDBY1702; row 4: BVGC16 & BVGC612; row 5: SDBY1705 and SDBY1706; row 6: SDBY1700 and SDBY1701) were serially diluted and spotted on synthetic complete agar plates supplemented with either 5 nM 5-Ad-IAA (*OsTIR1^F74A^* background) or 100 µM IAA (wild-type *OsTIR1* background) with or without 64 µg/mL fluconazole and grown for 2 days at 30°C. Note that untreated and fluconazole alone results are shown for the *OsTIR1^F74A^* background strains but were indistinguishable for the wild-type *OsTIR1* strains. (B) Serial dilution spotting assay of *C. glabrata* strains (row 1: KKY2001; row 2: BVGC16; rows 3–4: SDBY1703) with the indicated *OsTIR1* and *CDC14* genotypes on YPD with or without addition of 500 µM IAA. Two independent isolates of the CgCDC14-AID*/9xMyc strain (SDBY1703) were evaluated. Plates were grown for 2 days at 30°C prior to imaging.

*C. glabrata gcn5Δ* cells exhibit sensitivity to azole antifungal drugs (41), for example, in serial dilution plate spotting assays (Fig. 5A). GCN5-AID*/9xMyc cells were indistinguishable from untagged wild-type cells in the presence of fluconazole, confirming that the degron tag does not significantly impair Gcn5 function. In contrast, supplementation of fluconazole with 100 µM IAA in cells expressing wild-type *OsTIR1*, or 5 nM 5-Ad-IAA in cells expressing *OsTIR1^F74A^*, impaired growth of GCN5-AID*/9xMyc cells, consistent with reduced Gcn5 function. Interestingly, plating CgCDC14-AID*/9xMyc cells expressing wild-type OsTir1 on YPD plates containing 500 µM IAA completely prevented growth, suggesting that *CDC14* may be an essential gene in *C. glabrata*, as it is in *S. cerevisiae* (Fig. 5B). This is supported by a recent genome-wide transposon mutagenesis screen (42). Consistent with this, we have been unable to recover *cdc14Δ* transformants of *C. glabrata* using conventional methods. This highlights a key advantage of the AID system in studying essential genes. Collectively, our results demonstrate that the AID system in *C. albicans* and *C. glabrata* is robust enough to mimic gene deletion phenotypes and should be a useful tool for rapid target protein inactivation to allow phenotypic observation and functional characterization.

### Conclusions and guidelines

In this study, we demonstrated that AID can be successfully implemented in *Candida albicans* and *Candida glabrata* for the rapid and efficient degradation of diverse target proteins. The extent and speed of target protein degradation should make AID a useful functional tool for protein characterization in these species. We expect that the system will be readily adaptable to other fungal pathogens as well. AID offers several advantages over other common methods for protein functional characterization. Importantly, AID ensures normal system physiology until auxin is added, avoiding indirect effects of permanent genetic alterations like gene deletions, and longer-term transcriptional repression or RNAi systems. Moreover, AID maintains natural promoter control of the target gene, making it attractive for studying genes with highly regulated expression. The speed at which AID elicits target degradation makes it suitable for studying dynamic processes like signaling pathways, and for mimicking the effects of drugs. With the single requirement of exposing cells to auxin, AID is compatible with diverse experimental conditions, perhaps even live animal models of fungal infections or studies of fungal and host cell interactions in the future. Toward that end, current evidence suggests that AID2 system auxin analogs 5-Ad-IAA and the related 5-phenyl-IAA do not adversely affect growth of a variety of human cell cultures lines or mice (17, 24).

The plasmid reagents generated in this study were designed for testing and using AID technology in common lab strains of *C. albicans* and *C. glabrata* and are suitable for experimental use in any strains with the appropriate auxotrophic mutations, or in which the required auxotrophic mutations can be conveniently generated. To make AID useful for studying protein function in clinically derived pathogen strains and non-model species like the emergent *C. auris*, in which auxotrophic selection is not readily available, we are currently working on the expansion of our system to add recyclable antibiotic resistance markers for strain engineering. This may also make the system suitable for use in animal infection models where auxotrophic marker mutations can impact virulence (43, 44). The addition of N-terminal degron tag cassettes for targets that are not compatible with C-terminal fusions will be an important goal for future development as well.

The three target proteins we selected for testing AID technology behaved ideally, with consistent, extensive, and fast degradation observed specifically after auxin addition only in strains expressing OsTir1. Nonetheless, for each new target protein, it is prudent to optimize the auxin concentration, determine the kinetics and maximal percent degradation, and confirm the dependence of degradation on both Tir1 and auxin under the desired experimental conditions. As in other species, AID performance can vary with different target proteins because it functions by changing the equilibrium between protein synthesis and degradation. Differences in protein synthesis rates and mechanisms for regulating steady-state protein levels can result in different AID degradation kinetics and depletion levels. Researchers must also be aware that a degron tag may impair protein function or may not be accessible to OsTir1 and SCF due to protein structure or cellular localization effects. Modifications to AID to overcome problems with inefficient or auxin-independent degradation have been designed and validated in model organisms (40, 45 – 47) and may be feasible to implement in *Candida* species if needed.

## MATERIALS AND METHODS

### Plasmid construction

Plasmids constructed in this study are listed in Table S1. All pHLP plasmids were created using the In-Fusion cloning system (Takara Biosciences) and were confirmed by Wide-seq analysis. CTG clade codon-optimized coding sequences for (i) *OsTIR1* fused to 3xMyc and (ii) *3xV5/AID^F^* were synthesized by Twist Bioscience. pDIS3 (30), which has the targeting sequences for integration at the NEUT5L locus, was used as the starting point for pHLP710 assembly. An XhoI fragment containing the SAT1 marker was excised from pDIS3 and replaced with PCR-amplified *C. albicans URA3* from p347 (35), *C. albicans ACT1* promoter and intron cassette from pSFS2 (33), and codon-optimized synthetic *OsTIR1-3xMyc*. The In-Fusion assembly was designed so that the existing *TEF* terminator from pDIS3 followed the *OsTIR1-3xMyc* coding sequence. Site-directed mutagenesis (QuikChange II, Agilent) was used to introduce the Phe74 to Ala74 codon change in the *OsTIR1* coding sequence of pHLP710, creating pHLP712 for the AID2 system (17, 24). To create template plasmids for amplification of C-terminal degron tagging cassettes with the *SAT1* selectable marker we excised the existing degron/marker region from the original AID template plasmid pAR1070 (37) by digestion with NotI and HindIII and used In-Fusion assembly to insert (i) either the synthetic 3xV5/AID^F^ coding sequence (pHLP701) or AID*/3xHA sequence (pHLP700) amplified from pHyg-AID*-6HA (32) followed by (ii) the *S. cerevisiae ADH1* terminator, and (iii) the *ACT1* promoter*-SAT1* cassette with *C. albicans URA3* terminator amplified from pSFS2.

Plasmids, pJC608 and pJC609, are based on the pFA-Clox plasmid toolkit (31) and were constructed using the NEBuilder HiFi DNA Assembly Cloning kit (New England Biolabs) following manufacturer’s instructions. Primer design for PCR amplification of the different modules was performed with the NEBuilder Assembly tool (http://nebuilder.neb.com/). First, a 953 bp region from the *TDH3* promoter (−950 to +3) was amplified from genomic DNA and assembled into pFA-URA3-Clox vector digested with ClaI to produce the plasmid pFA-URA3-Clox-TDH3pr. Second, a 2120 bp fragment containing the *OsTIR1* gene and the TEF1 terminator sequence was amplified from pHLP710 or pHLP712 and assembled into the pFA-URA3-Clox-TDH3pr vector linearized with EcoRV to give rise to the pFA-URA3-Clox-TDH3pr-OsTIR1 (pJC608) and pFA-URA3-Clox-TDH3pr-OsTIR1^F74A^ (pJC609).

The plasmid pBV90 was constructed by first digesting the plasmid pBYP6744 (48) with EcoRI and XmaI to excise the *ADH1pr-OsTIR1* construct. By Gibson cloning (New England Biolabs), *ADH1pr-OsTIR1* was subsequently added into the pUC19 backbone along with Sc*LEU2* cassette and flanking regions of Cg*TRP1* for targeted insertion. pBV514 was constructed by the same strategy as pBV90 with the exception of the F74A mutation in the *OsTIR1* gene. The F74A mutation was constructed by Gibson cloning with overlapping primers harboring the desired mutation.

All plasmid constructs depicted in Fig. 1 are available through AddGene (https://www.addgene.org/, deposition #82873).

### Strain construction

Oligonucleotide primers used for strain constructions are listed in Table S2. All strains created or used in this study are listed in Table S3. For *OsTIR1* integration at *NEUT5L* in *C. albicans,* 25 µg pHLP710 or pHLP712 was digested with *SfiI,* ethanol-precipitated, and transformed by electroporation with selection on synthetic medium lacking uracil. Integration was confirmed by locus-specific PCR and anti-Myc immunoblotting. To integrate the *TDH3pr-OsTIR1^F74A^* cassette from pJC609 at *NEUT5L*, the *URA3*-Clox-*TDH3pr-OsTIR1^F74A^* module was amplified by PCR with primers S1-NEUT5L and S2-NEUT5L, and transformed by electroporation with selection for uracil prototrophy. Elimination of the *URA3* marker was performed as described (31). pBV90 and pBV514 were digested with PacI and SbfI, and transformed into *C. glabrata* (KKY2001) with selection for leucine prototrophy to make the strains BVGC16 and BVGC612, respectively.

Degron/epitope tags amplified from pHLP700 and pHLP701 were integrated at the 3ʹ end of the *CDC14* gene in *C. albicans* using PCR primers containing 21 template annealing bases and 69 bases of homology immediately upstream of the *CDC14* stop codon and downstream of a clustered regularly interspaced short palindromic repeats (CRISPR) guide RNA recognition site. Guide RNA was selected in the 3’ UTR as close to the stop codon as possible. The same strategy was used to tag *GCN5* and *CDC14* in *C. glabrata,* with the AID*/9xMyc degron tag amplified by PCR from pNAT-AID*−9Myc (32). PCR products (~1–2 µg) were ethanol-precipitated and resuspended in a small volume of sterile water and transformed by electroporation using a CRISPR-Cas9 RNP procedure described elsewhere (49) that was based on a previous report (50). The same CRISPR-Cas9 method was used to delete *GCN5* from *C. glabrata* using pAG25 as the PCR template. For tagging Mob2 at the C-terminus, cassettes were amplified from pHLP700 and pHLP701 using PCR primers listed in Table S2. PCR products were ethanol-precipitated and resuspended in 10 µL of sterile water and transformed by standard electroporation. Transformants were selected on YPD containing 300 µg/mL nourseothricin.

### Media and cell culture

Liquid cultures of *C. albicans* and *C. glabrata* were grown in YPD medium (10 g/L yeast extract, 20 g/L peptone, 20 g/L glucose) at 30°C with shaking at 225 rpm. Agar was added to 20% (wt/vol) for growth on solid media. For *C. albicans*’ *ura3* auxotrophic strains, YPD was supplemented with 8 µg/mL uridine. For agar plate spotting assays, liquid cultures started from single colonies were grown to saturation and serially diluted from starting OD_600_ = 0.125 in eightfold steps, with 5 µL of four consecutive dilutions spotted on plates. Plates were grown at 30°C for 3–5 days. *C. albicans cdc14Δ/Δ* spots were offset by one dilution step to normalize colony density on untreated control plates. For microplate growth assays, saturated cultures were diluted to optical density OD_600_ = 0.02 in YPD and mixed with an equal volume of YPD, YPD +2 mM IAA, or YPD +2 µM 5-Ad-IAA in a sterile, 96-well plate. Plates were incubated at 30°C with continuous orbital shaking at 425 cpm, measuring OD_600_ every 15 min for 24 h. Hyphal induction under embedded YPS (10 g/L yeast extract, 20 g/L peptone, 20 g/L sucrose) agar conditions was assessed as described previously (51). Hyphal induction was also monitored on Spider media agar plates (10 g/L beef broth, 10 g/L mannitol, 2 g/L K_2_HPO_4_, 20 g/L agar) grown at 37°C for up to 7 days. Auxin and stress agents at the indicated concentrations were added to cooled media immediately prior to pouring plates. MIC assays were performed based on the CLSI method for testing yeast, third edition (52). Briefly, yeast strains were inoculated in Roswell Park Memorial Institute (RPMI) medium and grown to saturation overnight, and then diluted to an OD_600_ of ~0.02 in RPMI with or without 2 mM IAA or 2 µM 5-Ad-IAA. Cells were then mixed 1:1 with a twofold dilution series of fluconazole or caspofungin in a 96-well polystyrene microplate. Plates were incubated at 35°C (for *C. glabrata*) or 30°C (for *C. albicans*), and MICs determined at 24 h based on the drug concentration where >90% of growth was inhibited.

For *C. glabrata* spotting assays, strains were inoculated in SC medium (Sunrise Science Products) and grown to saturation overnight. Cultures were diluted to an OD_600_ of 0.1 and grown in SC to log phase under shaking at 30°C. Each strain was spotted in fivefold dilutions starting at an OD_600_ of 0.01 on SC plates with or without the indicated concentrations of fluconazole (Cayman Chemical), 5-Ad-IAA, and IAA. Plates were grown at 30°C for 2–3 days and imaged.

For hyphal induction analysis in liquid medium, *C. albicans* strains were grown overnight to saturation in YPD and then diluted to OD_600_ = 0.5 in YPD + 10% serum supplemented with either 200 nM 5-Ad-IAA or an equal volume of DMSO at 37°C. Samples were collected 1 and 2 h later and imaged by DIC microscopy.

### SDS-PAGE and immunoblotting

Total protein extracts were prepared as described (35). Proteins were separated on 10% tris-glycine SDS-PAGE gels, transferred to 0.45 µm nitrocellulose membranes (Bio-Rad), and probed overnight at 4°C with mouse anti-HA (1:5,000; Sigma-Aldrich, 12CA5), rabbit anti-V5 (1:5,000; Invitrogen, MA5-15253), mouse anti-c-Myc (1:2,000–1:5,000; Sigma-Aldrich, 9E10), or rabbit anti-PSTAIR (1:5,000; Millipore-Sigma, 06-923). The rabbit polyclonal anti-H3 antibody was generated by Pocono Rabbit Farm & Laboratory and used at 1:100,000 dilution. Secondary anti-mouse and anti-rabbit antibodies conjugated to horseradish peroxidase were from Jackson ImmunoResearch (115-035-003 or 111-035-003) and used at 1:10,000 dilution for 60 min at 4°C. Immunoblots were developed with Clarity Western ECL Substrate (Bio-Rad, 170-5060) and imaged on a ChemiDoc MP multimode imager (Bio-Rad).

## SUPPLEMENTAL MATERIAL

Supplemental material msphere.00283-23-s0001.pdf Tables S1-S3 and Figures S1-S4.

ASM does not own the copyrights to Supplemental Material that may be linked to, or accessed through, an article. The authors have granted ASM a non-exclusive, world-wide license to publish the Supplemental Material files. Please contact the corresponding author directly for reuse.
