# Supplementary material for: Rapid, efficient auxin-inducible protein degradation in Candida pathogens
Source: mSphere. 2023 Aug 18;8(5):e00283-23. doi: 10.1128/msphere.00283-23 (PMC10597344; doi:10.1128/msphere.00283-23)
Supplement: Supplemental material — Tables S1-S3 and Figures S1-S4. [file msphere.00283-23-s0001.pdf]

SUPPLEMENTAL TABLES

Table S1. Plasmids created in this study

Name	Backbone	Yeast Marker	Expressed gene or tag
pHLP710	pDIS3 (1)	*URA3*	*ACT1pr-OsTIR1-3xMyc*
pHLP712	pDIS3 (1)	*URA3*	*ACT1pr-*$OsTIR1^{F74A}$*-3xMyc*
pHLP700	pAR1070 (2)	*SAT1*	AID*-3xHA
pHLP701	pAR1070 (2)	*SAT1*	3xV5-AID^{F}
pBV90	pUC19	*LEU2*	*ADH1pr-OsTIR1-9xMyc*
pBV514	pUC19	*LEU2*	*ADH1pr-*$OsTIR1^{F74A}$*-9xMyc*
pJC608	pFA-URA3-Clox (3)	*URA3*	*TDH3pr-OsTIR1*
pJC609	pFA-URA3-Clox (3)	*URA3*	*TDH3pr-*$OsTIR1^{F74A}$

Table S2. Oligonucleotides for strain engineering

**CRISPR Guide RNAs**	**Sequence (5' to 3')**
CaCDC14	AGUAUACAUUGAUUUAAUGA
CgGCN5-C-term Tag	GAGUUUGUAACAAUAUUUAG
CgGCN5-KO-5'	GAGAUUGCAUCACCCUGAAG
CgGCN5-KO-3'	GAAGAAUUUUUCAAGUCUGU
CgCDC14	AAGUAUAAAAUCGUUCUUAC
**Integrating Primers**	
S1-NEUT5L (#1213)	CCAATGGTGGTACCACTAACCCAAGAACAGAAAAAGCATTGATATTTTG<br>AAAAAATACAATACTCATCTTTTAACCCGAACCTTTTCTTGTCCTTTAAAA<br>CGAAGCTTCGTACGCTGCAGGTC
S2-NEUT5L (#1214)	CAATTATTAGAGATCCAGAAAACTGAATTGTGCTTGAATACCACTTGTTT<br>AGGAAGGACGATGAAGGAGAGAAAGTAGTAAGACAATATGACTTTGGA<br>CAGTCTGATATCATCGATGAATTCGAG
CalbCDC14_AID_CT_F	GCTTCTGGAAACTCACAAACATCAAGAGCACACTCTGGTGGTGTGAGA<br>AAGTTAAGTGGAAAGAAACATCGCTCTAGAACTAGTGGATCC
CalbCDC14_AID_CT_R	GAACCAGCTTATGAAGAAAACTAATTTAGAAAAAGTATAAATACGAAACA<br>AGTCTATAGTTTTACATACCCCTCGAGGTCGACGGTATCG
CgCDC14-AID*-F2	TCCATGACTAGCAATTATCAAAACTCCTCTTCTCAAAACAGAATTACAAG<br>GCAAACAACAGAAAGTGATGAAGAAGGTCTATTGAAGCAATTGTTACCA<br>AAGAATAGAAGAGTCGCATCAGGAAGAAGGAATGTCAGTGCTGCTGGT<br>GGTGTAAGAAAGGCCAGTGGTACAGTTAAAAGGCGTACGCTGCAGGTC<br>GAC
CgCDC14-AID*-R2	AGCTAAGAAATATAACGGCGTAAAACATAAGTCAGGCCATATCCTGATA<br>CCCTGTTATCTATGAGGTCTTGATTATTATCACCTCTATCGAAATATATA<br>CTCATTAACGTAAAATGGGTATTATTTGATTATTTGATTATATAACGAATG<br>CATATTTAATTAGAAGTTATTTGAAAACGCAATCGATGAATTCGAGCTCG
CgGCN5-AID*9MYC-F	TGGAGGAGTTTATTTACGACGCCAGATTAGTTTGTAACAACTGTCGGCT<br>GTATAATGGTGAGAACACTTCATATTACAAGTATGCCAACAGACTTGAA

	AAATTCTTCAATAATAAGGTTAAAGAAATACCTGAATACTCACATTTGATCGATCGTACGCTGCAGGTCGAC
CgGCN5-AID*9MYC-R	GATTCTTATACCAACTGGTTTTGTTTTGTATATATATTATGTGTCTATTTACCGTGTTTCTACATACTTAATGATAGAAATTTTGCATTTTTGAAAATGAGAGGCATCGATGAATTCGAGCTCG
CgGCN5-KO-F	AATGGGCGGTTAAAAAGCGTTTACACATTTCAGCTTTTGATTAGCACTGCAGCAAGTGTGTCATTAATTCATAGAGGCTTGTCGAGGCGAACAGTTTGAGTTGCAAGCACAAAAAAAAATAAATACTGGGTTTAACTAGCAAGAAACAACAAAGGAAAAATATAATCATGATGATTCATCCCATTCATTCCATC
CgGCN5-KO-R	ACTGGTTTTGTTTTGTATATATATTATGTGTCTATTTACCGTGTTTCTACATACTTAATGATAGAAATTTTGCATTTTTGAAAATGAGAGGATAATAATAATTTCATATATTTCATTAAGGATAATCCTCTAAATATTGTTACAAACTCAGAAGAATAGTACTATTGAATGCCGCTCTAGAACTAGTGGATCT
CaMOB2_AID-CT_F	TTCAATTTGATTGATAGAACTGAAATGGAACCGTTGTTACCTTTGATAGAGAATTTTGAACAACAAGGAAAAATCACCCAAGCAAGCAAACGCTCTAGAACTAGTGGATCC
CaMOB2_AID-CT_R	TTCATTCAGGCAATATACACGTACTATACTATACTATTCAATATATACACTAAACTCAACAATTCAAGGCACTAAATAAGATCAAATCTCGTAGTCTTGGCCCTCGAGGTCGACGGTATCG

PAM sites are not included in CRISPR gRNA sequences.
Underlining indicates template annealing sequences. Sequence without underlining reflects homology to target site.

Table S3. Yeast strains

Name	Genotype	Reference
C. albicans		
SC5314	Wild-type	
CAI4	*ura3::imm434/ura3::imm434*	(4)
BWP17	*ura3::imm434/ura3::imm434 his1::hisG/his1::his arg4::hisG/arg4::hisG*	(5)
JC2711	*CAI4 NEUT5L::URA3 cdc14::hisG/cdc14::hisG*	(6)
JC2712	*CAI4 NEUT5L::URA3*	(6)
JC2721	*CAI4 CDC14-3xHA:URA3/cdc14::hisG*	(6)
HCAL102	*CAI4* $cdc14^{(Q414A/P415A/K417A)}$*-3xHA:URA3/cdc14::hisG*	(6)
JC5	*CAI4 CDC14/cdc14::hisG*	(7)
HCAL110	*CAI4 CDC14/cdc14::hisG NEUT5L:OsTIR1-3xMyc:URA3*	This study
HCAL111	*CAI4 CDC14/cdc14::hisG NEUT5L:*$OsTIR1^{F74A}$*-3xMyc:URA3*	This study
HCAL112	*CAI4 CDC14-3xV5-*AID^{F}*:SAT1/cdc14::hisG*	This study
HCAL113	*CAI4 CDC14-AID*-3xHA:SAT1/cdc14::hisG*	This study
HCAL125	*CAI4 CDC14-3xV5-*AID^{F}*:SAT1/cdc14::hisG NEUT5L:OsTIR1-3xMyc:URA3*	This study
HCAL126	*CAI4 CDC14-3xV5-*AID^{F}*:SAT1/cdc14::hisG NEUT5L:*$OsTIR1^{F74A}$*-3xMyc:URA3*	This study
HCAL128	*CAI4 CDC14-AID*-3xHA:SAT1/cdc14::hisG NEUT5L:*$OsTIR1^{F74A}$*-3xMyc:URA3*	This study
JC2958	BWP17 *NEUT5L::loxP-TDH3p-OsTIR1-3xMyc*	This study
JC2960	BWP17 *NEUT5L::loxP-TDH3p-*$OsTIR1^{F74A}$*-3xMyc*	This study
OL3372	*BWP17 mob2::LUL/MOB2-3xV5-*AID^{F}*:SAT1; NEUT5L::loxP-TDH3p-*$OsTIR1^{F74A}$*-3xMyc*	This study
OL3309	*BWP17 mob2::loxP-MOB2-AID*-3xHA::SAT1; NEUT5L::URA3-ACT1p-*$OsTIR1^{F74A}$*-3xMyc*	This study
C. glabrata		
KKY2001	*his3::FRT leu2::FRT trp1::FRT*	(8)
BVGC16	*his3::FRT leu2::FRT trp1::ADH1p-OsTIR1-9xMyc:LEU2*	This study
BVGC612	*his3::FRT leu2::FRT trp1::ADH1p-*$OsTIR1^{F74A}$*-9xMyc:LEU2*	This study
SDBY1700	*his3::FRT leu2::FRT trp1::ADH1p-OsTIR1-9xMyc:LEU2 GCN5-AID*-9xMyc*	This study
SDBY1701	*his3::FRT leu2::FRT trp1::ADH1p-*$OsTIR1^{F74A}$*-9xMyc:LEU2 GCN5-AID*-9xMyc*	This study
SDBY1702	*his3::FRT leu2::FRT trp1::FRT GCN5-AID*-9xMyc*	This study
SDBY1703	*his3::FRT leu2::FRT trp1::ADH1p-OsTIR1-9xMyc:LEU2 CDC14-AID*-9xMyc:*	This study
SDBY1704	*his3::FRT leu2::FRT trp1::FRT gcn5::NatMX*	This study
SDBY1705	*his3::FRT leu2::FRT trp1::ADH1p-OsTIR1-9xMyc:LEU2 gcn5::NatMX*	This study
SDBY1706	*his3::FRT leu2::FRT trp1::ADH1p-*$OsTIR1^{F74A}$*-9xMyc:LEU2 gcn5::NatMX*	This study

SUPPLEMENTAL FIGURE LEGENDS

Figure S1. AID technology provides rapid, efficient target degradation in *C. albicans*. A-C) Repeat trials for experiments shown in Figure 2 panels A-C. Percent protein remaining relative to the untreated culture was quantified by digital imaging. **D-F)** Repeat trials for Figure 2 panels D-F showing degradation kinetics of Cdc14 proteins in response to IAA (D) or 5-Ad-IAA (E-F) treatment. Percent protein remaining relative to time=0 was quantified by digital imaging. These values, combined with those from Figure 2, were used to calculate half-life from a simple exponential decay function using GraphPad Prism.

Figure S2. AID technology provides rapid, efficient target degradation in *C. glabrata*. A-B) Repeat trials for experiments shown in Figure 3 panels A-B. Percent protein remaining relative to the untreated culture was quantified by digital imaging. **C-D)** Repeat trials for Figure 3 panels C-D showing degradation kinetics of Gcn5 proteins in response to IAA (C) or 5-Ad-IAA (D) treatment. Percent protein remaining relative to time=0 was quantified by digital imaging. These values, combined with those from Figure 3, were used to calculate half-life from a simple exponential decay function using GraphPad Prism.

Figure S3. IAA and 5-Ad-IAA do not affect proliferation, stress sensitivity, or hyphal growth of *C. albicans*. A) Liquid cultures of *C. albicans* with the indicated genotypes (SC5314, HCAL110, and HCAL111 from left to right) were back-diluted to OD_{600} = 0.01 in YPD in a 96-well microplate, supplemented with the indicated concentrations of IAA or 5-Ad-IAA, and grown at 30°C with shaking for 24 hours in a plate reader, measuring absorbance at 600 nm. **B)** A liquid culture of *C. albicans* (CAI4) was serially diluted and spotted on YPD agar plates supplemented with the indicated chemicals in the absence or presence of 1 mM IAA or 1 μM 5-Ad-IAA and grown at 30 °C for 3 days prior to imaging. Concentrations of H_2O_2, MMS, NaCl,

and fluconazole were chosen slightly lower than those causing noticeable toxicity compared to the “No Stress” plates. **C)** Same as panel B with *C. glabrata* strain KKY2001 and with micafungin replacing fluconazole. Images were taken after 2 days growth at 30 °C. Qualitatively identical results were obtained with KKY2001 expressing *OsTIR1* or *OsTIR1(F74A)*, not shown. **D)** Liquid cultures of *C. albicans* BWP17 were transferred to YPD + 10% serum at 37 °C to induce hyphal growth in the absence or presence of 50 nM 5-Ad-IAA and hyphal length was measured at several timepoints. Mean lengths were plotted as a function of serum induction time on the right. **E)** Standard liquid minimum inhibitory concentration (MIC) assays in RPMI for fluconazole and caspofungin were conducted with *C. albicans* strain SC5314 in the presence and absence of the indicated auxins. Green color represents relative growth, measured as absorbance at 600 nm (average of 3 biological replicates) in each well after 24 hours. Similar results were obtained with *C. glabrata* strain KKY2001, with and without expression of *OsTIR1*.

Figure S4. AID effectively phenocopies loss-of-function mutations in *C. albicans*. The indicated *C. albicans* strains (JC2712, HCAL126, JC2711, HCAL102 from top to bottom) were grown as individual colonies on Spider agar plates at 37 °C for 7 days prior to imaging. All images represent identical plate areas.

REFERENCES

1. Gerami-Nejad M, Zacchi LF, McClellan M, Matter K, Berman J. 2013. Shuttle vectors for facile gap repair cloning and integration into a neutral locus in Candida albicans. Microbiology 159:565–579.

2. Powers BL, Hall MC. 2017. Re-examining the role of Cdc14 phosphatase in reversal of Cdk phosphorylation during mitotic exit. J Cell Sci 130:2673–2681.

3. Dueñas-Santero E, Santos-Almeida A, Rojo-Dominguez P, Rey F del, Correa-Bordes J, Vázquez de Aldana CR. 2019. A new toolkit for gene tagging in Candida albicans containing recyclable markers. PLoS One 14:e0219715.

4. Fonzi WA, Irwin MY. 1993. Isogenic strain construction and gene mapping in Candida albicans. Genetics 134:717–728.

5. Wilson BR, Davis D, Mitchell AP. 1999. Rapid hypothesis testing with Candida albicans through gene disruption with short homology regions. J Bacteriol 181:1868–1874.

6. Milholland KL, AbdelKhalek A, Baker KM, Hoda S, DeMarco AG, Naughton NH, Koeberlein AN, Lorenz GR, Anandasothy K, Esperilla-Muñoz A, Narayanan SK, Correa-Bordes J, Briggs SD, Hall MC. 2023. Cdc14 phosphatase contributes to cell wall integrity and pathogenesis in Candida albicans. Front Microbiol 14.

7. Clemente-Blanco A, Gonzalez-Novo A, Machin F, Caballero-Lima D, Aragon L, Sanchez M, Vázquez de Aldana CR, Jimenez J, Correa-Bordes J. 2006. The Cdc14p phosphatase affects late cell-cycle events and morphogenesis in Candida albicans. J Cell Sci 119:1130–43.

8. Paul S, McDonald WH, Moye-Rowley WS. 2018. Negative regulation of Candida glabrata Pdr1 by the deubiquitinase subunit Bre5 occurs in a ubiquitin independent manner. Mol Microbiol 110:309–323.

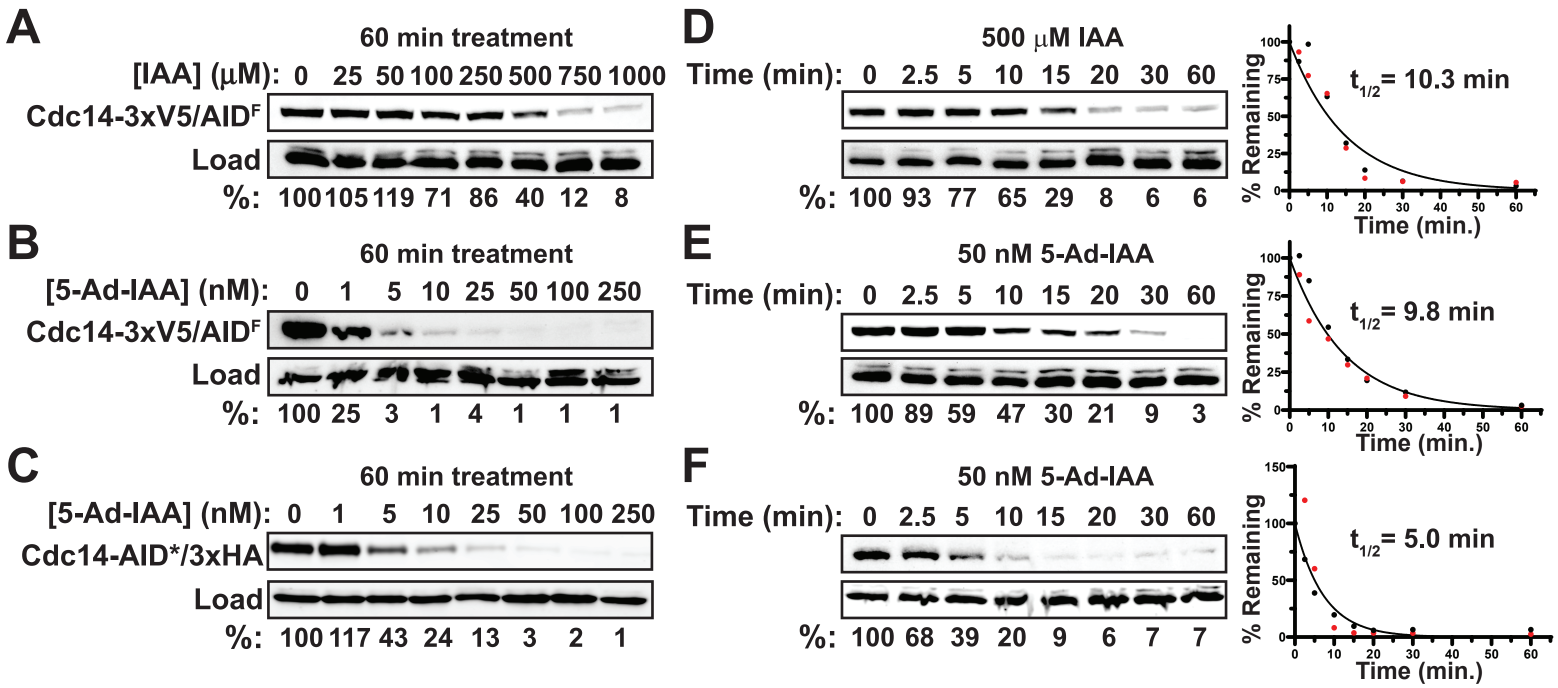
A
60 min treatment
[IAA] (μM): 0 25 50 100 250 500 750 1000
Cdc14-3xV5/AID^F
Load
%: 100 105 119 71 86 40 12 8
B
60 min treatment
[5-Ad-IAA] (nM): 0 1 5 10 25 50 100 250
Cdc14-3xV5/AID^F
Load
%: 100 25 3 1 4 1 1 1
C
60 min treatment
[5-Ad-IAA] (nM): 0 1 5 10 25 50 100 250
Cdc14-AID*/3xHA
Load
%: 100 117 43 24 13 3 2 1
D
500 μM IAA
Time (min): 0 2.5 5 10 15 20 30 60
%: 100 93 77 65 29 8 6 6
t1/2= 10.3 min
% Remaining
Time (min.)
E
50 nM 5-Ad-IAA
Time (min): 0 2.5 5 10 15 20 30 60
%: 100 89 59 47 30 21 9 3
t1/2= 9.8 min
% Remaining
Time (min.)
F
50 nM 5-Ad-IAA
Time (min): 0 2.5 5 10 15 20 30 60
%: 100 68 39 20 9 6 7 7
t1/2= 5.0 min
% Remaining
Time (min.)

Figure S1

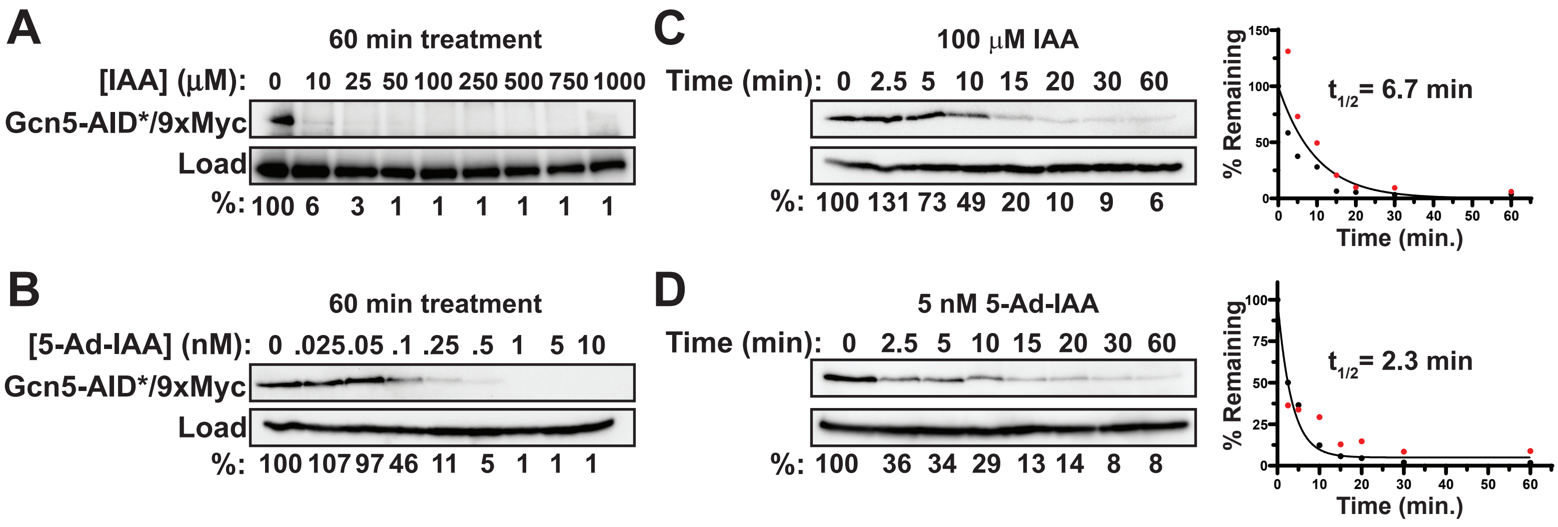
A
60 min treatment
[IAA] (μM): 0 10 25 50 100 250 500 750 1000
Gcn5-AID*/9xMyc
Load
%: 100 6 3 1 1 1 1 1 1
B
60 min treatment
[5-Ad-IAA] (nM): 0 .025 .05 .1 .25 .5 1 5 10
Gcn5-AID*/9xMyc
Load
%: 100 107 97 46 11 5 1 1 1
C
100 μM IAA
Time (min): 0 2.5 5 10 15 20 30 60
%: 100 131 73 49 20 10 9 6
% Remaining
t1/2= 6.7 min
Time (min.)
D
5 nM 5-Ad-IAA
Time (min): 0 2.5 5 10 15 20 30 60
%: 100 36 34 29 13 14 8 8
% Remaining
t1/2= 2.3 min
Time (min.)

Figure S2

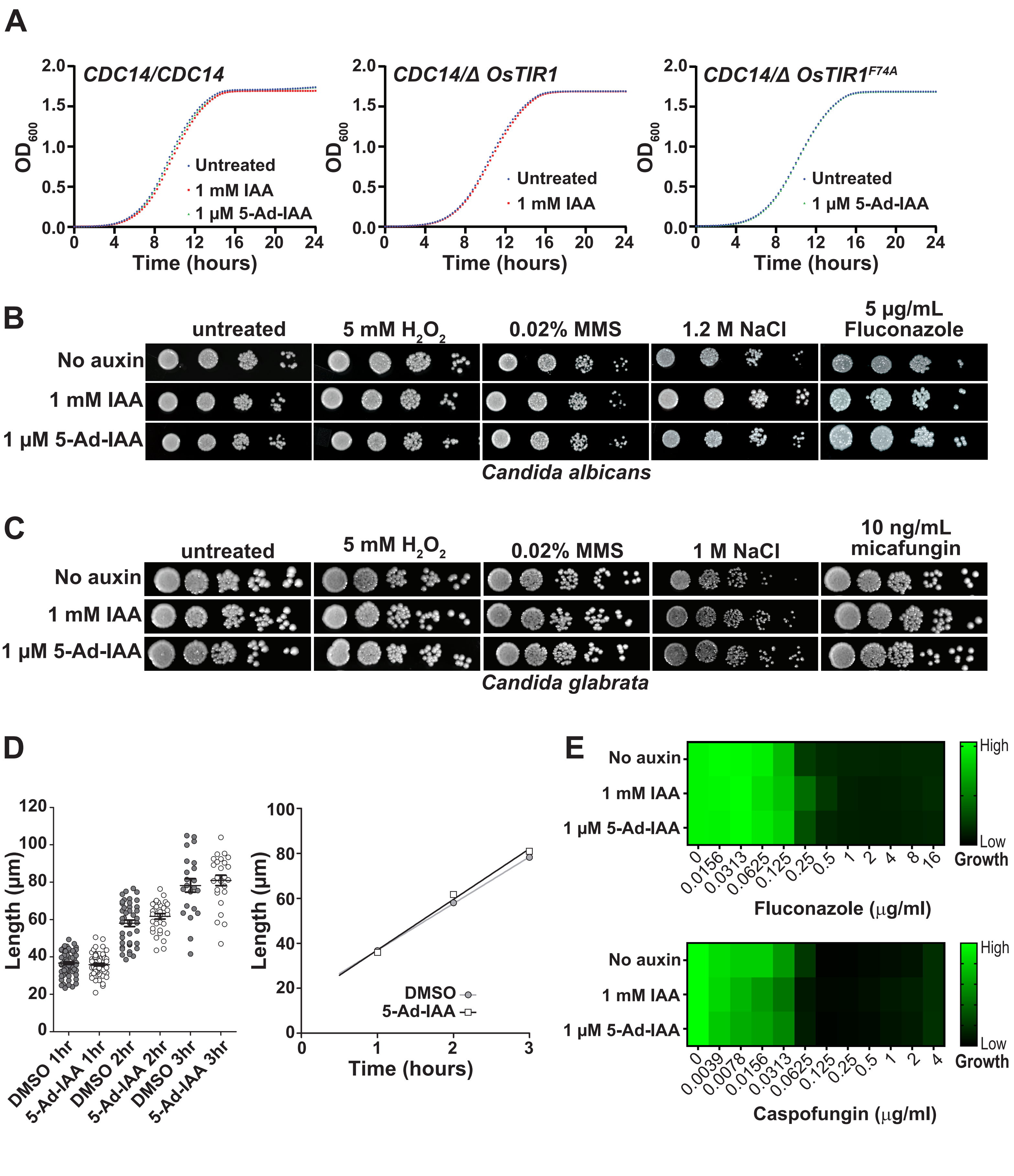
A
CDC14/CDC14
CDC14/Δ OsTIR1
CDC14/Δ OsTIR1F74A
OD600
Untreated
1 mM IAA
1 μM 5-Ad-IAA
Time (hours)
B
untreated
5 mM H2O2
0.02% MMS
1.2 M NaCl
5 μg/mL Fluconazole
No auxin
1 mM IAA
1 μM 5-Ad-IAA
Candida albicans
C
untreated
5 mM H2O2
0.02% MMS
1 M NaCl
10 ng/mL micafungin
No auxin
1 mM IAA
1 μM 5-Ad-IAA
Candida glabrata
D
Length (μm)
DMSO 1hr
5-Ad-IAA 1hr
DMSO 2hr
5-Ad-IAA 2hr
DMSO 3hr
5-Ad-IAA 3hr
DMSO
5-Ad-IAA
Time (hours)
E
No auxin
1 mM IAA
1 μM 5-Ad-IAA
High
Low
Growth
Fluconazole (μg/ml)
0 0.0156 0.0313 0.0625 0.125 0.25 0.5 1 2 4 8 16
Caspofungin (μg/ml)
0 0.0039 0.0078 0.0156 0.0313 0.0625 0.125 0.25 0.5 1 2 4

Figure S3

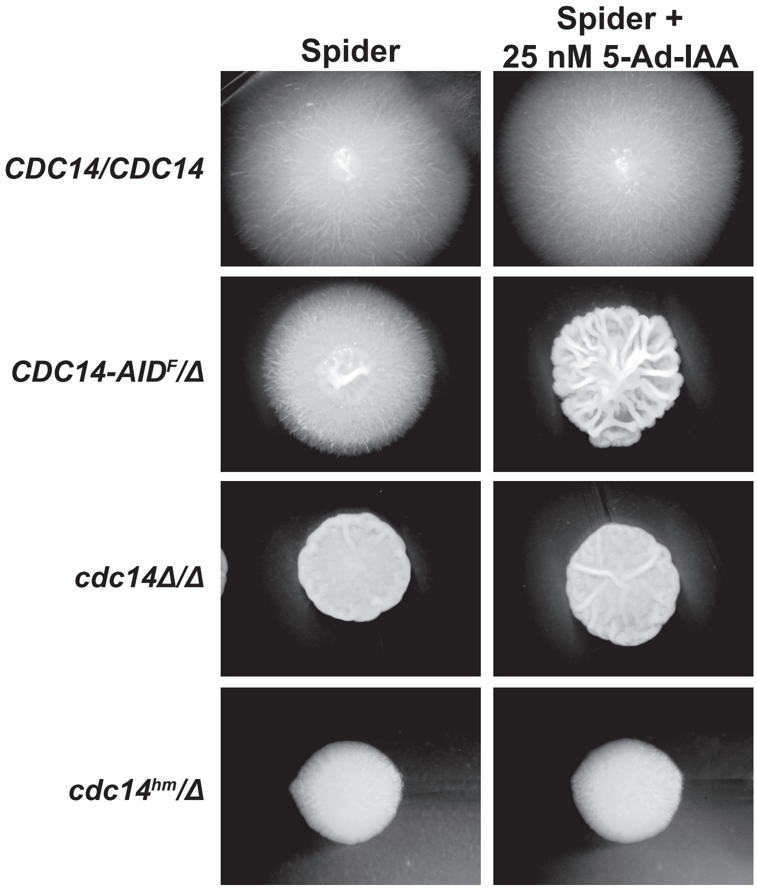
Spider
Spider +
25 nM 5-Ad-IAA
CDC14/CDC14
CDC14-AID^F/Δ
cdc14Δ/Δ
cdc14^hm/Δ
